# Association of Paraoxonase-1 (p.L55M) and Paraoxonase-2 (p.S311C) polymorphisms with coronary artery disease in North Indian Punjabi population

**DOI:** 10.3389/fendo.2025.1688319

**Published:** 2025-10-07

**Authors:** Mohd Akbar Bhat, Jatinder Singh, Ghulam Mohd Lone, Shiwali Goyal

**Affiliations:** ^1^ Multidisciplinary Research Unit, Government Medical College, Amritsar, Punjab, India; ^2^ Department of Ophthalmology, Georgetown University Medical Center, Washington DC, United States; ^3^ Department of Pharmacology, Government Medical College, Amritsar, Punjab, India; ^4^ Department of Pharmacology, Dr. Radhakrishnan Government Medical College, Hamirpur, Himachal Pradesh, India; ^5^ Ophthalmic Genetics and Visual Function Branch, National Eye Institute, National Institutes of Health, Bethesda, MD, United States

**Keywords:** coronary artery disease, PON1, PON2, polymorphisms, haplotype, North India

## Abstract

**Background:**

Paraoxonases (PONs) are a unique family of calcium-dependent enzymes which are tightly associated with the high-density lipoprotein cholesterol (HDL-C), plays a crucial role in protecting the low-density lipoprotein cholesterol (LDL-C) from oxidation, thereby providing protection against atherosclerosis-a key factor for the pathogenesis of coronary artery disease (CAD). The activity of PON enzymes is influenced by genetic polymorphisms in the *PON* genes. The present case-control study was performed to investigate the association of *PON1* (p.L55M, rs854560) and *PON2* (p.S311C, rs7493) polymorphisms with CAD in the North Indian Punjabi population.

**Methods and results:**

The present study included 211 CAD patients and 260 healthy controls genotyped using the polymerase chain reaction-reaction fragment length polymorphism (PCR-RFLP) technique. Binary logistic regression analysis revealed that the SC and CC genotypes of the *PON2* (p.S311C) conferred 2-and 3.5-folds increased risk for CAD (OR: 2.03, 95%CI: 1.36-3.01, p=0.001; OR: 3.49, 95%CI: 1.86-6.55, p=0.001, respectively). Moreover, the dominant (OR: 2.29, 95%CI: 1.58-3.32, p=0.0001), co-dominant (OR: 1.62, 95%CI: 1.11-2.36, p=0.012), recessive (OR: 2.58, 95%CI: 1.41-4.72, p=0.001), and log-additive (OR: 1.92, 95%CI: 1.46-2.54, p=0.0001) are the best-fit inheritance models to predict the susceptible gene effects. Furthermore, the LC haplotype (PON1 and PON2) was found to be significantly and independently associated with the increased risk of CAD (OR: 2.34, 95%CI: 1.65-3.32, p=0.0001).

**Conclusions:**

Our results indicate a significant and independent association of *PON2* (p.S311C) polymorphism with CAD even after gender stratification in North Indian Punjabi population.

## Introduction

Coronary artery disease (CAD) is a widespread and complex condition with a multifactorial etiology, where both genetic predispositions and environmental influences, along with their interactions, are critical in its pathogenesis (1). It is one of the leading causes of both morbidity and mortality globally (2). Cardiovascular diseases (CVDs) account for 31% of global deaths, with CAD responsible for approximately 7.4 million of these fatalities (3). Individuals from the South Asian subcontinent experience a higher mortality rate from CAD compared to other ethnic groups (4). The primary traditional risk factors for CAD include lipid abnormalities, diabetes, hypertension, smoking, obesity, gender, advanced age, and a family history of the disease (5). Additionally, oxidative modification of low-density lipoprotein cholesterol (LDL-C) is crucial in the formation of atherosclerotic plaques, which contributes to the onset and progression of CAD (5). Conversely, high-density lipoprotein cholesterol (HDL-C) provides a protective effect against atherosclerosis by preventing LDL oxidation (6), and is inversely related to CAD development (7). The protective role of HDL-C is attributed to its associated enzymes, such as paraoxonase (PON), platelet-activating factor acetylhydrolase, and lecithin-cholesterol acyltransferase, with PON being the most extensively studied (8). Paraoxonases (PONs) are a distinct group of calcium-dependent hydrolases and esterases located on the HDL surface that protect LDL from oxidation and possess antiatherogenic, antioxidative, and anti-inflammatory properties (9). PON activity is notably affected by genetic polymorphisms, making PON variants potential biomarkers for CAD due to their influence on LDL oxidation (10).

The paraoxonase (PON) gene family comprises *PON1*, *PON2*, and *PON3*, located on the long arm of chromosome 7 (7q21.3-q22.1) (11). Among these, *PON1* is a key susceptibility gene involved in vascular pathology and is considered a promising biomarker for CAD. It is a 44-kDa glycoprotein enzyme that depends on calcium and is primarily produced in the liver, where it associates with the surface of high-density lipoproteins. In the bloodstream, PON1, which is predominantly linked to HDL, inhibits LDL oxidation, promotes cellular cholesterol efflux from macrophages (12), and reduces lipid peroxides within atherosclerotic plaques (13). *PON1* exhibits several polymorphisms (14), with the p.L55M (rs854560) variant being particularly well-studied across different ethnic groups for its impact on CAD susceptibility (15). The leucine-to-methionine substitution at codon 55 (p.L55M) results in reduced stability and has been linked to decreased PON1 levels and activity (16). Lower PON1 activity contributes to the initiation and progression of atherosclerotic plaques and exacerbates the severity of related atherosclerosis-related conditions (17).

Human paraoxonase 2 (*PON2*) is a member of the paraoxonase gene family, known for its distinct anti-oxidative and anti-atherosclerotic properties. PON2 is an intracellular protein that is ubiquitously expressed, particularly in the human heart, endothelial cells, and aortic smooth muscle cells (18). It plays a critical role in preventing LDL oxidation, reversing the oxidation of mildly oxidized LDL, and protecting against atherosclerosis (19). Moreover, PON2 expression in macrophages increases under oxidative stress, suggesting that PON2 may serve as a selective antioxidant at the cellular level, thereby playing an anti-atherogenic role by reducing macrophage foam cell formation and oxidative stress (20). Additionally, PON2’s anti-apoptotic function contributes significantly to atherosclerotic protection (21). Given its role in preventing LDL oxidation, similar to PON1, PON2 has become a focus of extensive research to understand its contribution to CAD susceptibility across different ethnic groups. Various polymorphisms in *PON2* have been studied in relation to different diseases. Among them, the p.S311C (rs7493) polymorphism has been widely investigated for its association with CAD development across many ethnic populations, where it is considered an important risk factor for CAD (9, 22–25). Notably, no studies from India have yet reported an association between the *PON2* (p.S311C) polymorphism and CAD. The substitution of serine to cysteine at codon 311 (p.S311C) in *PON2* (26) results in decreased enzyme activity, thereby reducing the protection of LDL from oxidation (27). Consequently, abnormal paraoxonase activity due to different genotypes may contribute to the higher incidence of CAD in individuals over 50 years of age (22). Population-based studies have shown inter-ethnic differences in allele frequencies for *PON1* (p.L55M) and *PON2* (p.S311C) polymorphisms. This variability indicates that ethnic differences, gene-gene interactions, and environmental susceptibility might influence the relationship between *PON* polymorphisms and CAD. These findings support the role of impaired PON1 activity in atherosclerosis and strengthen the rationale for investigating PON polymorphisms in CAD. Therefore, considering the significance of PON polymorphisms in CAD genetic susceptibility and the variation in allele frequencies among different ethnic groups, the present case-control study was designed. It aimed to explore the association of *PON1* (p.L55M) and *PON2* (p.S311C) polymorphisms with CAD in the North Indian Punjabi population.

## Materials and methods

### Patients and controls

The current study involved 211 patients diagnosed with CAD based on electrocardiographic (ECG) findings (such as ST-segment depression/elevation or T-wave inversions consistent with angina), echocardiographic alterations (including regional wall motion abnormalities or evidence of impaired left ventricular function consistent with ischemic heart disease), and positive results from treadmill testing (defined as ≥1 mm horizontal or downsloping ST-segment depression or elevation during exercise accompanied by typical anginal symptoms). These patients were recruited from the Government Medical College, Amritsar, with exclusions made for individuals with liver, lung, kidney, or thyroid disorders, as well as those with malignancies. The control group consisted of 260 healthy individuals from the general population, matched for age, sex, and ethnicity, with no current or past family history of CAD or other diseases. The study received approval from the Ethics Committee of Government Medical College, Amritsar, and adhered to the principles outlined in the Declaration of Helsinki. Clinical data, including age, gender, height, weight, body mass index (BMI), hip and waist circumference, and family history, were collected for all participants using a pre-designed proforma. All participants provided voluntary written informed consent. Blood pressure was measured using a mercury sphygmomanometer, following the American Heart Association’s standard procedures (28). A trained technician collected 5 ml of whole blood from each participant after an overnight fast, with 3 ml transferred into EDTA vials for DNA extraction and the remaining 2 ml into vials without anticoagulant for serum separation. Biochemical parameters, including total cholesterol (TC), triglycerides (TG), and high-density lipoprotein cholesterol, were measured using a semi-automated biochemical analyzer (Erba Chem-7) with commercially available kits (Erba). Low-density lipoprotein cholesterol and very-low-density lipoprotein cholesterol (VLDL-C) levels were calculated using Friedewald’s formula (29).

### Genotyping

DNA was extracted from peripheral blood leukocytes using the phenol-chloroform method (30). Genotyping of the *PON1* (p.L55M, rs854560) and *PON2* (p.S311C, rs7493) variants was conducted through polymerase chain reaction-restriction fragment length polymorphism (PCR-RFLP). The primer sequences used were as follows: for *PON1*, forward: 5’-GAAGAGTGATGTATAGCCCCAG-3’ and reverse: 5’-ACACTCACAGAGCTAATGAAAGCC-3’; for *PON2*, forward: 5’-ACATGCATGTACGGTGGTCTTATA-3’ and reverse: 5’-AGCAATTCATAGATTAATTGTTA-3’. The PCR reactions were performed in a 15 µl total volume containing 50 ng of genomic DNA, 1.5 mM MgCl_2_, 0.2 mM of each dNTP, 10 μM of each primer, and 1 U of Taq DNA polymerase using a Mastercycler gradient thermal cycler (Eppendorf, Hamburg, Germany). The cycling conditions included an initial denaturation at 95°C for 5 minutes, followed by 35 cycles of denaturation at 95°C for 45 seconds, annealing at 52°C for *PON1* (p.L55M) and 51°C for *PON2* (p.S311C) for 45 seconds, extension at 72°C for 45 seconds, and a final extension at 72°C for 10 minutes. The amplified PCR products for *PON1* (171 bp) and *PON2* (265 bp) were digested with *Nla*III and *Dde*I restriction enzymes (New England Biolabs, USA) respectively and then visualized on a 3% agarose gel stained with ethidium bromide. For the *PON1* polymorphism, the undigested 171 bp fragment represents the L allele, while the 127 bp and 44 bp fragments correspond to the M allele. In the *PON2* polymorphism, the 123 bp, 75 bp, and 67 bp fragments indicate the S allele, whereas the 142 bp and 123 bp fragments correspond to the C allele. To ensure genotyping accuracy, positive controls with known genotypes were included in each run, and 10% of samples per SNP were randomly re-genotyped to validate accuracy.

### Statistical analysis

Data analyses were conducted using the Statistical Package for Social Sciences, version 31 (SPSS Inc., Chicago, IL, USA). Continuous variables were presented as mean ± standard deviation and analyzed using Student’s t-test. Categorical variables were reported as counts and percentages, with differences between categorical variables and genotype distributions assessed using the Chi-squared test with Yates correction. Comparisons of clinical and lipid parameters across different genotypes were performed using one-way ANOVA followed by Tukey’s *post hoc* test. Hardy-Weinberg equilibrium, haplotype analysis, and genetic models (dominant, co-dominant, recessive, and log-additive) were evaluated using SNPstats software. Binary logistic regression analysis was employed to calculate odds ratios (ORs) and 95% confidence intervals (CIs) for different *PON1* (p.L55M) and *PON2* (p.S311C) genotypes to assess CAD risk, both before and after adjusting for potential covariates such as age, gender, BMI, waist circumference, hypertension, and alcohol consumption. Power calculations were performed using the CaTS-Power Calculator (31) with the study achieving a statistical power of 90% at a significance level of 0.05 to detect an association with an OR of 1.5. A p-value of <0.05 was considered statistically significant, and for multiple comparisons, p-values were adjusted using the Bonferroni correction.

## Results

### Baseline characteristics of the study population

The baseline characteristics of the CAD patients and controls are shown in **Table 1**. CAD patients have significantly (p=0.001) higher values for BMI, waist circumference (WC), waist-hip ratio (WHR), waist-height ratio (WHtR), systolic blood pressure (SBP), and diastolic blood pressure (DBP) compared to healthy controls. Moreover, lipid levels (TC, TG, LDL-C, VLDL-C) were significantly (p=0.001) higher, while pulse pressure (PP) and HDL-C levels were significantly (p=0.001) lower in CAD patients than in controls. Gender-specific analysis shows that all variables, except for WHR in males were significantly (p=0.001) higher in CAD patients, with PP and HDL-C being significantly (p=0.001) lower in both male and female patients compared to their respective controls (**Supplementary Table 1**).

**Table 1:** Demographic and clinical characteristics of the study participants.

Variables	Patients (n=211)	Controls (n=260)	P-value
Age (years)	57.84 ± 11.33	57.33 ± 12.97	0.650
Gender (male/female)	101/110	129/131	0.776
Alcohol consumption (yes/no)	37/174	59/201	0.205
BMI (kg/m^2^)	26.87 ± 4.97	24.87 ± 4.62	0.001
WC (cm)	99.55 ± 10.69	94.62 ± 9.77	0.001
WHR	1.01 ± 0.06	0.99 ± 0.06	0.001
WHtR	0.61 ± 0.07	0.58 ± 0.06	0.001
SBP (mmHg)	133.70 ± 17.99	119.00 ± 8.13	0.001
DBP (mmHg)	85.11 ± 10.77	78.85 ± 6.43	0.001
PP	48.59 ± 11.57	79.73 ± 27.49	0.001
TC (mg/dL)	288.03 ± 35.50	189.40 ± 35.97	0.001
TG (mg/dL)	229.94 ± 69.33	96.14 ± 37.51	0.001
HDL-C (mg/dL)	24.69 ± 5.28	50.39 ± 12.85	0.001
LDL-C (mg/dL)	216.15 ± 37.50	119.49 ± 39.34	0.001
VLDL-C (mg/dL)	47.23 ± 17.22	19.52 ± 8.67	0.001

BMI, body mass index; WC, waist circumference; WHR, waist-to-hip ratio; WHtR, waist-to-height ratio; SBP, systolic blood pressure; DBP, diastolic blood pressure; PP, pulse pressure; TC, total cholesterol; TG, triglycerides; HDL-C, high-density lipoprotein-cholesterol; LDL-C, low-density lipoprotein cholesterol; VLDL-C, very low-density lipoprotein cholesterol.

Bonferroni corrected p=0.003.

### Genotype distribution in CAD patients and healthy controls

The genotype and allele frequency distributions of *PON1* (p.L55M) and *PON2* (p.S311C) polymorphisms are detailed in **Table 2**. The genotype distribution of *PON1* and *PON2* polymorphisms in both cases and controls were in agreement with those predicted by the Hardy-Weinberg equilibrium (p<0.05). The frequencies of SC and CC genotypes of *PON2* (p.S311C) were significantly (p=0.0001) higher in CAD patients (43.1%, 16.1%) than in controls (31.9%, 6.9%), respectively. The C allele was observed to be more frequent in patients (37.7%) than in controls (22.9%). After gender stratification, the frequencies of SC and CC genotypes were observed to be significantly higher in male (43.6%, 18.8%) and female (42.7%, 13.6%) patients compared to their respective controls (34.9%, 7.7%) and (29%, 6.1%). The C allele was also more common in male and female patients (40.6%, 35%) than in corresponding controls (25.2%, 20.6%) (**Supplementary Table 2**). However, the genotype and allele distributions of *PON1* (p.L55M) were not statistically significant in total (p=0.641, p=0.601) (**Table 2**) or when stratified by gender (0.907, p=0.915 in males), and (p=0.442, p=0.601 in females) (**Supplementary Table 2**). All observed associations remained persistent even after Bonferroni correction (p=0.025). This lack of association suggests that the *PON1* (p.L55M) polymorphism may not play a major role in CAD susceptibility in the North Indian Punjabi population. It also highlights the potential influence of ethnic and genetic heterogeneity on the contribution of *PON1* variants to CAD risk.

**Table 2:** Genotype and allele distributions of *PON1* (L55M) and *PON2* (S311C) polymorphisms in the study participants.

	CAD patients (n=211)	Controls (n=260)	χ^2^	P-value
Genotypes*PON1* (L55M)				
LL	131 (62.1%)	165 (63.5%)	0.888	0.641
LM	68 (32.2%)	85 (32.7%)		
MM	12 (5.7%)	10 (3.8%)		
L allele	330 (78.2%)	415 (79.8%)	0.274	0.601
M allele	92 (21.8%)	105 (20.2%)		
Genotypes*PON2* (S311C)				
SS	86 (40.8%)	159 (61.2%)	22.184	0.0001
SC	91 (43.1%)	83 (31.9%)		
CC	34 (16.1%)	18 (6.9%)		
S allele	263 (62.3%)	401 (77.1%)	23.800	0.001
C allele	159 (37.7%)	119 (22.9%)		

### Association between *PON* polymorphisms and CAD

The association results of *PON1* and *PON2* polymorphisms are presented in **Table 3**. Binary logistic regression analysis revealed that the SC and CC genotypes of *PON2* polymorphism exhibited 2-and 3.5-folds increased risk for CAD susceptibility (OR: 2.03, 95%CI: 1.36-3.01, p=0.001; OR: 3.49, 95%CI: 1.86-6.55, p=0.001, respectively). Even after adjusting for potential covariates such as age, gender, BMI, WC, hypertension and alcohol consumption, the SC and CC genotypes continued to demonstrate a 1.9-and 3.7-folds increased risk for CAD (OR: 1.96, 95%CI: 1.25-3.08, p=0.004; OR: 3.68, 95%CI: 1.84-7.33, p=0.001, respectively). In addition, the dominant (OR: 2.29, 95%CI: 1.58-3.32, p=0.0001), co-dominant (OR: 1.62, 95%CI: 1.11-2.36, p=0.012), recessive (OR: 2.58, 95%CI: 1.41-4.72, p=0.001), and log-additive (OR: 1.92, 95%CI: 1.46-2.54, p=0.0001) models have also shown significant association with the increased risk for CAD. These associations remained significant even after adjustment, the dominant (OR: 2.26, 95%CI: 1.48-3.45, p=0.0001), co-dominant (OR: 1.53, 95%CI: 1.00-2.23, p=0.051), recessive (OR: 2.75, 95%CI: 1.42-5.31, p=0.002), and log-additive (OR: 1.93, 95%CI: 1.42-2.63, p=0.0001).

**Table 3:** Logistic regression analysis between *PON1* (L55M) and *PON2* (S311C) polymorphisms and CAD risk in the study participants.

	Patients (n=211)	Controls (n=260)	OR (95%CI)	P-value	^a^OR (95%CI)	P-value
Genotypes, genetic models*PON1* (L55M)						
LL	131 (62.1%)	165 (63.5%)	Ref	–	–	–
LM	68 (32.2%)	85 (32.7%)	1.01 (0.68-1.49)	0.969	1.03 (0.66-1.61)	0.890
MM	12 (5.7%)	10 (3.8%)	1.51 (0.63-3.61)	0.352	1.39 (0.53-3.60)	0.501
L allele frequency	330 (78.2%)	415 (79.8%)	Ref		–	–
M allele frequency	92 (21.8%)	105 (20.2%)	1.11 (0.80-1.51)	0.546		
Dominant model (LL vs. LM+MM)	–	–	1.06 (0.73-1.54)	0.759	1.07 (0.70-1.64)	0.750
Co-dominant model (LM vs. LL+MM)	–	–	0.98 (0.66-1.44)	0.915	1.01 (0.65-1.56)	0.980
Recessive model (MM vs. LL+LM)	–	–	1.51 (0.64-3.58)	0.349	1.37 (0.54-3.51)	0.510
Log-additive model	–	–	1.10 (0.81-1.50)	0.550	1.10 (0.77-1.56)	0.610
Genotypes, genetic models*PON2* (S311C)						
SS	86 (40.8%)	159 (61.2%)	Ref	–	–	–
SC	91 (43.1%)	83 (31.9%)	2.03 (1.36-3.01)	0.001	1.96 (1.25-3.08)	0.004
CC	34 (16.1%)	18 (6.9%)	3.49 (1.86-6.55)	0.001	3.68 (1.84-7.33)	0.001
S allele frequency	263 (62.3%)	401 (77.1%)	Ref		–	–
C allele frequency	159 (37.7%)	119 (22.9%)	2.04 (1.53-2.71)	0.001		
Dominant model (SS vs. SC+CC)	–	–	2.29 (1.58-3.32)	0.0001	2.26 (1.48-3.45)	0.0001
Co-dominant model (SC vs. SS+CC)	–	–	1.62 (1.11-2.36)	0.012	1.53 (1.00-2.35)	0.051
Recessive model (CC vs. SS+SC)	–	–	2.58 (1.41-4.72)	0.001	2.75 (1.42-5.31)	0.002
Log-additive model	–	–	1.92 (1.46-2.54)	0.0001	1.93 (1.42-2.63)	0.0001

SNPs, single nucleotide polymorphisms; OR, odds ratio; CI, confidence interval.

a OR adjusted for age, gender, BMI, WC, hypertension and alcohol consumption.

Bonferroni corrected p = 0.025 (p = 0.05/number of SNPs).

On gender stratification, the SC (OR: 1.90, 95%CI: 1.08-3.37, p=0.027) and CC (OR: 3.70, 95%CI: 1.57-8.74, p=0.003) genotypes showed 1.9-and 3.7-folds increased risk for CAD in males and after adjustment, the SC genotype lost its risk (OR: 1.60, 95%CI: 0.84-3.04, p=0.151) while the CC genotype continued to exhibit a 3.9-fold increased risk for CAD (OR: 3.93, 95%CI: 1.51-10.22, p=0.005). Moreover, the dominant (OR: 2.23, 95%CI: 1.31-3.80, p=0.003), recessive (OR: 2.76, 95%CI: 1.22-6.23, p=0.012), and log-additive (OR: 1.92, 95%CI: 1.30-2.83, p=0.0008) models have also indicated increased risk for CAD. After adjustment, the dominant (OR: 1.98, 95%CI: 1.09-3.59, p=0.023), recessive (OR: 3.19, 95%CI: 1.29-7.91, p=0.011), and log-additive (OR: 1.86, 95%CI: 1.21-2.87, p=0.004) models continued to show significant and independent associations with the increased risk for CAD (**Supplementary Table 3**). Similarly, the SC and CC genotypes exhibited 2.2-and 3.3-folds increased risk for CAD in females (OR: 2.19, 95%CI: 1.26-3.82, p=0.006; OR: 3.32, 95%CI: 1.31-8.40, p=0.011, respectively) and after adjustment, the risk increased 2.5-fold for the SC genotype (OR: 2.52, 95%CI: 1.32-4.82, p=0.005) and 4.1-folds for the CC genotype (OR: 4.10, 95%CI: 1.46-11.55, p=0.008). In addition, the dominant (OR: 2.39, 95%CI: 1.42-4.02, p=0.0009), co-dominant (OR: 1.83, 95%CI: 1.07-3.11, p=0.026), recessive (OR: 2.43, 95%CI: 0.99-5.96, p=0.047), and log-additive (OR: 1.96, 95%CI: 1.31-2.96, p=0.0007) models also indicated an increased risk for CAD. After adjustment, these models continued to show significant and independent associations with the increased risk of CAD, the dominant (OR: 2.79, 95%CI: 1.03-7.50, p=0.042), co-dominant (OR: 1.99, 95%CI: 1.08-3.68, p=0.028), recessive (OR: 2.78, 95%CI: 1.03-7.50, p=0.028), and log-additive (OR: 2.19, 95%CI: 1.38-3.46, p=0.0006) (**Supplementary Table 4**). There were no differences in statistical significance even after the Bonferroni correction (p=0.025).

### Clinical characteristics of the study participants stratified by genotypes

The clinical characteristics of the study participants stratified according to different genotypes of *PON1* (p.L55M) and *PON2* (p.S311C) are shown in **Table 4**. No significant differences for clinical variables were found among different genotypes, neither in the total group nor after gender stratification (**Supplementary Tables 5**, **6**).

**Table 4:** Distribution of clinical parameters in different genotypes of *PON1* (L55M) and *PON2* (S311C) in the study participants.

Variables	*PON1* (L55M)				*PON2* (S311C)			
	LL (n=131)	LM (n=68)	MM (n=12)	P-value	SS (n=86)	SC (n=91)	CC (n=34)	P-value
Age (years)	57.31 ± 11.22	58.38 ± 11.23	60.50 ± 13.49	0.579	56.58 ± 11.00	57.60 ± 11.96	59.88 ± 10.28	0.319
BMI (kg/m^2^)	26.50 ± 4.72	27.53 ± 5.37	27.19 ± 5.34	0.377	26.84 ± 5.54	26.88 ± 4.56	26.92 ± 4.61	0.997
WC (cm)	98.70 ± 10.61	100.64 ± 10.68	102.63 ± 11.48	0.285	99.49 ± 11.41	99.87 ± 9.84	98.82 ± 11.29	0.886
WHR	1.01 ± 0.06	1.02 ± 0.05	1.01 ± 0.05	0.325	1.02 ± 0.06	1.01 ± 0.06	1.01 ± 0.06	0.545
WHtR	0.60 ± 0.07	0.62 ± 0.07	0.64 ± 0.08	0.175	0.61 ± 0.07	0.61 ± 0.06	0.61 ± 0.07	0.490
SBP (mmHg)	133.28 ± 18.58	134.70 ± 16.75	132.69 ± 19.53	0.854	134.05 ± 16.49	133.77 ± 20.59	132.62 ± 14.21	0.925
DBP (mmHg)	85.16 ± 11.42	84.81 ± 9.49	86.39 ± 10.97	0.895	84.87 ± 10.12	85.74 ± 10.99	84.08 ± 11.90	0.720
PP	48.13 ± 11.45	49.89 ± 12.02	46.30 ± 10.37	0.467	49.19 ± 11.12	48.04 ± 12.65	48.55 ± 9.75	0.805
TC (mg/dL)	286.19 ± 32.35	290.31 ± 42.09	295.10 ± 27.91	0.577	290.87 ± 40.88	284.37 ± 29.45	290.59 ± 35.86	0.431
TG (mg/dL)	235.53 ± 77.16	217.22 ± 50.76	241.11 ± 64.72	0.178	238.35 ± 68.65	222.54 ± 70.70	228.48 ± 66.87	0.316
HDL-C (mg/dL)	24.81 ± 5.19	24.20 ± 5.59	26.23 ± 4.39	0.433	24.57 ± 5.12	24.43 ± 5.41	25.70 ± 5.39	0.471
LDL-C (mg/dL)	213.45 ± 37.61	220.55 ± 38.03	220.65 ± 33.09	0.411	217.38 ± 42.51	214.58 ± 33.53	217.20 ± 34.87	0.871
VLDL-C (mg/dL)	48.00 ± 17.53	45.56 ± 17.34	48.22 ± 12.94	0.627	48.91 ± 16.86	45.36 ± 16.79	47.98 ± 19.18	0.376

BMI, body mass index; WC, waist circumference; WHR, waist-to-hip ratio; WHtR, waist-to-height ratio; SBP, systolic blood pressure; DBP, diastolic blood pressure; PP, pulse pressure; TC, total cholesterol; TG, triglycerides; HDL-C, high-density lipoprotein-cholesterol; LDL-C, low-density lipoprotein cholesterol; VLDL-C, very-low-density lipoprotein cholesterol.

Bonferroni corrected p=0.003.

### Linkage disequilibrium and haplotype analysis

**Figure 1:**
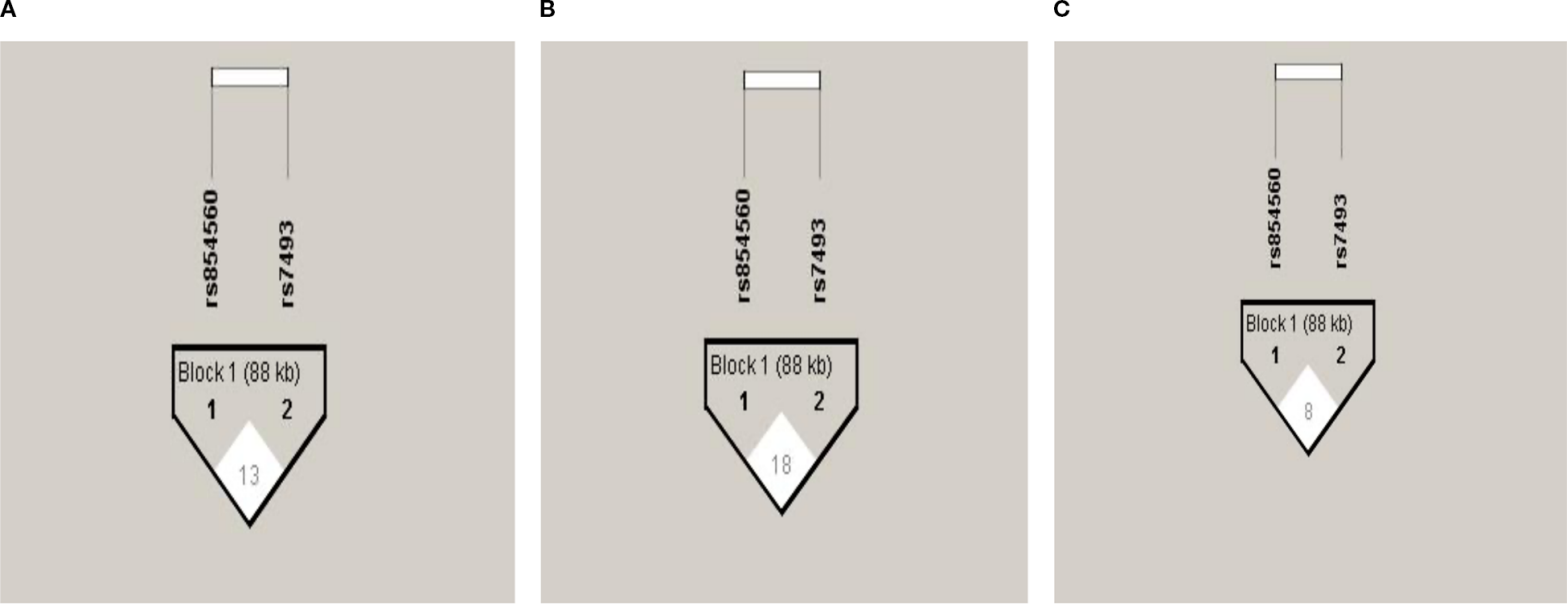
Pair-wise Linkage disequilibrium plot of PON1 (p.L55M) and PON2 (p.S311C) SNPs in the total group **(A)**, males **(B)**, and females **(C)**. The hatch marks contain D’ values multiplied by 100 to indicate the strength of LD between the SNPs.

The *PON1* (p.L55M) and *PON2* (p.S311C) SNPs were not in LD in the total group (**Figure 1A**) or after gender stratification (**Figures 1B, C**) despite being located within the same gene cluster. Haplotype analysis (**Table 5**) revealed that the LC haplotype showed a 2.3-fold increased risk for CAD (OR: 2.34, 95%CI: 1.65-3.32, p=0.0001) and after adjustment, this risk remained at 2.2-fold (OR: 2.22, 95%CI: 1.50-3.29, p=0.0001). Gender-specific analysis showed that the LC haplotype conferred a 2.5-fold increased risk for CAD in males (OR: 2.52, 95% CI: 1.54-4.13, p=0.0003) and a 2.2-fold increased risk in females (OR: 2.22, 95% CI: 1.34-3.67, p=0.002) (**Supplementary Table 7**). After adjustment, the risk remained significant, with a 2.2-fold increase in males (OR: 2.21, 95% CI: 1.29-3.80, p=0.004) and a 2.4-fold increase in females (OR: 2.41, 95% CI: 1.36-4.27, p=0.003). The associations remained persistent after the Bonferroni correction (p=0.0125).

**Table 5:** Haplotype analysis of *PON1* (L55M) and *PON2* (S311C) polymorphisms in the study population.

	PON1	PON2	OR (95%CI)	P-value	^a^OR (95%CI)	P-value
1	L	S	1.00	–	1.00	–
2	L	C	2.34 (1.65-3.32)	0.0001	2.22 (1.50-3.29)	0.0001
3	M	S	1.17 (0.97-2.24)	0.067	1.35 (0.84-2.16)	0.210
4	M	C	1.33 (0.66-2.71)	0.430	1.57 (1.76-3.28)	0.230

Global Haplotype association p-value <0.0001.

OR, odds ratio; CI, confidence interval.

a OR adjusted for age, gender BMI, WC, hypertension and alcohol consumption.

Bonferroni corrected p = 0.0125 (p-value = 0.05/number of haplotypes).

## Discussion

Genetic susceptibility to CAD is influenced by the involvement of numerous genes that participate in metabolic pathways related to the pathogenesis and progression of atherosclerosis (32). Among these, the genes encoding paraoxonase-1 (*PON1*) and paraoxonase-2 (*PON2*) have been identified as contributing to genetic susceptibility to CAD. Polymorphisms in *PON1* (p.L55M) and *PON2* (p.S311C) are known to affect PON activity. Various studies have explored the association between these polymorphisms and their impact on CAD risk (33). *In vivo* animal studies have highlighted the anti-atherogenic properties of PON1 and PON2. Tward et al. (34) demonstrated that transgenic mice with elevated expression of the human *PON1* gene produce high-density lipoproteins with enhanced anti-oxidative properties, offering protection against atherosclerosis compared to wild-type mice. Additionally, PON1-deficient mice have been found to have an increased risk of developing atherosclerosis compared to their wild-type counterparts (35). Similarly, PON2 has been shown to protect against low-density lipoprotein oxidation and inhibit monocyte transmigration in response to LDL oxidation in PON2-knockout mice (19). Interestingly, these PON2-knockout mice exhibit an increased number of foam cells and lipid droplets, along with significantly larger atherosclerotic lesions compared to wild-type mice (36). Previous studies indicate that PON2 can mitigate atherogenesis and reduce atherosclerotic lesions (37). These findings suggest that *PON1* and P*ON2* polymorphisms may play a role in modulating atherogenesis, a critical factor in the initiation and progression of CAD. Consequently, we conducted a case-control study to investigate the association between *PON1* (p.L55M) and *PON2* (p.S311C) polymorphisms and CAD in the North Indian Punjabi population.

In this cohort, there was a statistically significant difference in the genotypic distribution of the *PON2* (p.S311C) polymorphism between CAD patients and controls (p=0.0001), with a higher prevalence of the CC genotype in CAD patients (16.1%) compared to controls (6.9%). The SC and CC genotypes were associated with a 2-fold and 3.5-fold increased risk of developing CAD, respectively. After adjusting for potential confounding factors, the SC genotype remained associated with a 2-fold increased risk, while the CC genotype conferred a 3.7-fold increased risk. These findings indicate that the (p.S311C) polymorphism is significantly and independently linked to an elevated risk of CAD in the North Indian Punjabi population. When stratified by gender, the SC and CC genotypes were found to increase CAD risk by 1.9-fold and 3.7-fold in males, and by 2.2-fold and 3.3-fold in females, respectively, suggesting that both males and females carrying the 311Cys allele are more susceptible to developing CAD. These findings align with those of Jalilian et al. (38) and Ding et al. (17), who reported a significant association between the 311Cys allele and CAD in Iranian and Taiwanese populations, respectively. Additionally, Elnoamany et al. (24) documented the association of the 311Cys allele with CAD in the Egyptian population. In line with our findings, a recent study from a Polish cohort demonstrated that the PON2 311S allele was independently associated with a higher risk of complex type C coronary lesions, highlighting its potential role in lesion severity and CAD progression (39). However, our results differ from those of Sanghera et al. (22) and Pan et al. (9) who found an association between the 311Ser allele and CAD in Asian Indians (Singapore) and Taiwanese populations. Guxens et al. (23) also reported an association between the 311Ser allele and acute myocardial infarction in a Spanish population. Furthermore, studies in other ethnic groups, including Japanese, Brazilian, and Polish populations, have shown no association between the *PON2* (p.S311C) polymorphism and CAD (40–42).

For the *PON1* (p.L55M) polymorphism, no significant differences were observed in the genotype and allele frequency distributions between CAD cases and controls (p=0.641; p=0.601, respectively). Our findings are consistent with those of Munshi et al. (33), Aruljothi et al. (43), and Godbole et al. (44) who also reported no significant association between the *PON1* (p.L55M) polymorphism and CAD in North and South Indian populations. Similarly, Gupta et al. (45) and Kaur et al. (46) found no significant association between the *PON1* (L55M) polymorphism and CAD in North-West Indian Punjabis and Asian Indians, respectively. Other studies have also shown no association between the *PON1* (p.L55M) polymorphism and CAD in North Indian, Turkish, South Indian and Polish populations (8, 39, 47, 48). A recent meta-analysis further supports the lack of a significant association between the *PON1* (p.L55M) polymorphism and CAD (49). However, our results differ from those of Oliveira et al. (41) who identified a protective effect of the M allele of *PON1* against CAD in a Brazilian population. Similarly, other studies reported a significant association between the *PON1* (p.L55M) polymorphism and CAD in Australian, Turkish, Iranian and Bulgarian populations (15, 50–52). These discrepancies across different populations may be due to ethnic heterogeneity, population diversity, varying environmental and genetic backgrounds, differences in sample sizes, and diverse study designs (53). Additionally, association studies alone may not be sufficient to determine the role of specific SNPs, particularly in polymorphic genes where many SNPs are in LD.

In this study, we did not observe any significant differences in clinical parameters across different genotypes of *PON1* (p.L55M) and *PON2* (p.S311C) among CAD cases, including after gender-based analysis, consistent with previous findings (24, 46). Haplotype analysis revealed that the LC haplotype was significantly associated with an increased risk of developing CAD, even after adjusting for potential covariates. Furthermore, gender stratification indicated that the LS haplotype was linked to a higher risk for CAD in both males and females, both before and after adjustment. These findings suggest that *PON2* modulates CAD risk independently and also synergistically with *PON1* in the overall patient group, including when stratified by gender, in the North Indian Punjabi population. Previous studies have also suggested that *PON2* may work synergistically with *PON1* polymorphisms, independent of other risk factors (22).

In our cohort, the significant association of the *PON2* (p.S311C) polymorphism with CAD suggests a possible genotype-phenotype correlation, as this variant has been implicated in modulating oxidative stress pathways that contribute to atherosclerotic progression. In contrast, the absence of association for the *PON1* (p.L55M) polymorphism highlights the potential population-specific variability in genotype effects. Clinically, such findings underscore the importance of considering genetic diversity when assessing CAD risk and suggest that PON polymorphisms may serve as adjunctive markers for personalized risk stratification in the future.

The study entails some strengths and limitations. To the best of our knowledge, this is the first study that reported a significant and independent association of *PON2* (p.S311C) polymorphism with CAD in the North Indian Punjabi population. The study also confirmed the interactive effect of *PON2* and *PON1* variants with CAD, implying that *PON2* individually or in combination with *PON1* plays a role in modulating CAD risk. However, there are some limitations to consider. The primary limitation was the small sample size, highlighting the need for more ethnic studies with larger sample sizes to confirm these results. Secondly, absence of enzyme activity measurements of PON1 and PON2 weakens the ability to directly establish the mechanistic link between genotype and phenotype. Hence, future studies integrating both genetic and functional analyses are necessary to better elucidate the biological role of these polymorphisms in CAD. Lastly, the study focused on only two SNPs, future research should analyze a broader range of SNPs together with functional characterization to better establish the role of these polymorphisms as risk factors for CAD.

## Conclusion

The study reported a significant and independent association of *PON2* (p.S311C) polymorphism with the increased risk of CAD in the North Indian Punjabi population even after being stratified by gender. To better understand the role of *PON2* in CAD susceptibility, further research is needed across various ethnic populations, so that early preventive strategies could be initiated to manage and mitigate disease progression.

## Data Availability

The original contributions presented in the study are included in the article/**Supplementary Material**. Further inquiries can be directed to the corresponding author/s.
