# Supplementary material for: Association of Paraoxonase-1 (p.L55M) and Paraoxonase-2 (p.S311C) polymorphisms with coronary artery disease in North Indian Punjabi population
Source: Front Endocrinol (Lausanne). 2025 Oct 7;16:1688319. doi: 10.3389/fendo.2025.1688319 (PMC12537402; doi:10.3389/fendo.2025.1688319)
Supplement: Supplementary file 1 [file SupplementaryFile1.docx]

**Supplementary Table 1. Demographic and clinical characteristics of the male and female study participants**

| **Variables** | **Male patients**  **(n=101)** | **Male controls (n=129)** | **p-value** | **Female patients (n=110)** | **Female controls (n=131)** | **p-value** |
| --- | --- | --- | --- | --- | --- | --- |
| **Age (years)** | 57.79±11.2 | 58.73±12.21 | 0.546 | 57.88±11.49 | 55.95±13.57 | 0.234 |
| **Alcohol consumption (yes/no)** | 37/64 | 59/70 | 0.209 | 0/110 | 0/131 | - |
| **BMI (kg/m^2^)** | 26.49±4.14 | 24.81±4.22 | 0.001 | 27.22±5.62 | 24.95±4.99 | 0.001 |
| **WC (cm)** | 98.94±10.25 | 94.81±9.61 | 0.001 | 100.11±11.09 | 94.42±9.95 | 0.001 |
| **WHR** | 1.02±0.05 | 1.01±0.05 | 0.273 | 1.01±0.06 | 0.99±0.07 | 0.001 |
| **WHtR** | 0.58±0.06 | 0.56±0.05 | 0.001 | 0.63±0.07 | 0.60±0.07 | 0.001 |
| **SBP (mmHg)** | 134.05±18.41 | 120.04±8.02 | 0.001 | 133.39±17.69 | 117.99±8.14 | 0.001 |
| **DBP (mmHg)** | 84.48±10.54 | 79.42±6.60 | 0.001 | 85.78±10.99 | 78.30±6.24 | 0.001 |
| **PP** | 49.57±11.59 | 76.70±30.63 | 0.001 | 47.69±11.54 | 82.73±23.73 | 0.001 |
| **TC (mg/dL)** | 295.03±40.89 | 189.20±36.01 | 0.001 | 281.59±28.41 | 189.59±36.07 | 0.001 |
| **TG (mg/dL)** | 223.81±64.40 | 93.20±40.15 | 0.001 | 235.57±73.40 | 99. 02±34.67 | 0.001 |
| **HDL-C (mg/dL)** | 23.86±4.85 | 50.76±13.39 | 0.001 | 25.46±5.56 | 50.02±12.32 | 0.001 |
| **LDL-C (mg/dL)** | 224.31±39.35 | 119.09±39.93 | 0.001 | 208.66±34.21 | 119.89±38.91 | 0.001 |
| **VLDL-C (mg/dL)** | 46.96±18.93 | 19.35±10.03 | 0.001 | 47.48±15.56 | 19.68±7.12 | 0.001 |

BMI: body mass index; WC: waist circumference; WHR: waist-to-hip ratio; WHtR: waist-to-height ratio; SBP: systolic blood pressure; DBP: diastolic blood pressure; PP: pulse pressure; TC: total cholesterol; TG: triglycerides; HDL-C: high-density lipoprotein-cholesterol; LDL-C: low-density lipoprotein cholesterol; VLDL-C: very-low-density lipoprotein cholesterol.

Bonferroni corrected p=0.003

**Supplementary Table 2. Gender-wise genotype and allele distributions of *PON1* (L55M) and *PON2* (S311C) polymorphisms**

|  | **Male patients (n=101)** | **Male controls (n=129)** | **χ^2^** | **p-value** |
| --- | --- | --- | --- | --- |
| **Genotypes *PON1* (L55M)** | | | | |
| LL | 62 (61.4%) | 82 (63.6%) | 0.192 | 0.907 |
| LM | 34 (33.7%) | 40 (31%) |  |  |
| MM | 5 (4.9%) | 7 (5.4%) |  |  |
| L allele | 158 (78.2%) | 204 (79.1%) | 0.011 | 0.915 |
| M allele | 44 (21.8%) | 54 (20.9%) |  |  |
| **Genotypes *PON2* (S311C)** | | | | |
| SS | 38 (37.5%) | 74 (57.4%) | 11.132 | 0.004 |
| SC | 44 (43.6%) | 45 (34.9%) |  |  |
| CC | 19 (18.8%) | 10 (7.7%) |  |  |
| S allele | 120 (59.4%) | 193 (74.8%) | 11.659 | 0.001 |
| C allele | 82 (40.6%) | 65 (25.2%) |  |  |
|  | **Female patients**  **(n=110)** | **Female controls (n=131)** | **χ^2^** | **p-value** |
| **Genotypes *PON1* (L55M)** | | | | |
| LL | 69 (62.7%) | 83 (63.4%) | 1.634 | 0.442 |
| LM | 34 (30.9%) | 45 (34.4%) |  |  |
| MM | 7 (6.4%) | 3 (2.3%) |  |  |
| L allele | 172 (78.2%) | 211 (80.5%) | 0.274 | 0.601 |
| M allele | 48 (21.8%) | 51 (19.5%) |  |  |
| **Genotypes *PON2* (S311C)** | | | | |
| SS | 48 (43.6%) | 85 (64.9%) | 11.635 | 0.003 |
| SC | 47 (42.7%) | 38 (29%) |  |  |
| CC | 15 (13.6%) | 8 (6.1%) |  |  |
| S allele | 143 (65%) | 208 (79.4%) | 11.794 | 0.001 |
| C allele | 77 (35%) | 54 (20.6%) |  |  |

**Supplementary Table 3. Logistic regression analysis between *PON1* (L55M) and *PON2* (S311C) polymorphisms and CAD risk in males**

|  | **Male patients**  **(n=101)** | **Male controls (n=129)** | **OR (95%CI)** | **p-value** | **^a^OR (95%CI)** | **p-value** |
| --- | --- | --- | --- | --- | --- | --- |
| **Genotypes, genetic models *PON1* (L55M)** | | | | | | |
| LL | 62 (61.4%) | 82 (63.6%) | **Ref** | - | - | - |
| LM | 34 (33.7%) | 40 (31%) | 1.12 (0.64-1.97) | 0.684 | 1.24 (0.66-2.33) | 0.490 |
| MM | 5 (4.9%) | 7 (5.4%) | 0.94 (0.29-3.12) | 0.926 | 0.68 (0.17-2.69) | 0.586 |
| L allele frequency | 158 (78.2%) | 204 (79.1%) | **Ref** |  | - | - |
| M allele frequency | 44 (21.8%) | 54 (20.9%) | 1.05 (0.67-1.65) | 0.825 |  |  |
| Dominant model  (LL vs. LM+MM) | - | - | 1.10 (0.64-1.88) | 0.736 | 1.15 (0.63-2.09) | 0.660 |
| Co-dominant model  (LM vs. LL+MM) | - | - | 1.05 (0.68-1.63) | 0.828 | 1.28 (0.69-2.38) | 0.440 |
| Recessive model  (MM vs. LL+LM) | - | - | 0.91 (0.28-2.95) | 0.872 | 0.64 (0.16-2.46) | 0.440 |
| Log-additive model | - | - | 1.05 (0.68-1.63) | 0.830 | 1.03 (0.63-1.68) | 0.910 |
| **Genotypes, genetic models *PON2* (S311C)** | | | | | | |
| SS | 38 (37.5%) | 74 (57.4%) | **Ref** | - | - | - |
| SC | 44 (43.6%) | 45 (34.9%) | 1.90 (1.08-3.37) | 0.027 | 1.60 (0.84-3.04) | 0.151 |
| CC | 19 (18.8%) | 10 (7.7%) | 3.70 (1.57-8.74) | 0.003 | 3.93 (1.51-10.22) | 0.005 |
| S allele frequency | 120 (59.4%) | 193 (74.8%) | **Ref** |  | - | - |
| C allele frequency | 82 (40.6%) | 65 (25.2%) | 2.03 (1.36-3.02) | 0.001 |  |  |
| Dominant model  (SS vs. SC+CC) | - | - | 2.23 (1.31-3.80) | 0.003 | 1.98 (1.09-3.59) | 0.023 |
| Co-dominant model  (SC vs. SS+CC) | - | - | 1.44 (0.84-2.46) | 0.180 | 1.21 (0.66-2.20) | 0.540 |
| Recessive model  (CC vs. SS+SC) | - | - | 2.76 (1.22-6.23) | 0.012 | 3.19 (1.29-7.91) | 0.011 |
| Log-additive model | - | - | 1.92 (1.30-2.83) | 0.0008 | 1.86 (1.21-2.87) | 0.004 |

SNPs: single nucleotide polymorphisms; OR= odds ratio; CI= confidence interval

^a^OR adjusted for age, BMI, WC, hypertension and alcohol consumption

Bonferroni corrected p = 0.025 (p = 0.05 / number of SNPs)

**Supplementary Table 4. Logistic regression analysis between *PON1* (L55M) and *PON2* (S311C) polymorphisms and CAD risk in females**

|  | **Female patients**  **(n=110)** | **Female controls (n=131)** | **OR (95%CI)** | **p-value** | **^a^OR (95%CI)** | **p-value** |
| --- | --- | --- | --- | --- | --- | --- |
| **Genotypes, genetic models *PON1* (L55M)** | | | | | | |
| LL | 69 (62.7%) | 83 (63.4%) | **Ref** | - | - | - |
| LM | 34 (30.9%) | 45 (34.4%) | 0.91 (0.52-1.57) | 0.733 | 0.88 (0.46-1.67) | 0.688 |
| MM | 7 (6.4%) | 3 (2.3%) | 2.81 (0.69-11.26) | 0.146 | 2.65 (0.59-11.89) | 0.203 |
| L allele frequency | 172 (78.2%) | 211 (80.5%) | **Ref** |  | - | - |
| M allele frequency | 48 (21.8%) | 51 (19.5%) | 1.15 (0.74-1.79) | 0.524 |  |  |
| Dominant model  (LL vs. LM+MM) | - | - | 1.03 (0.61-1.74) | 0.919 | 1.00 (0.54-1.85) | 0.998 |
| Co-dominant model  (LM vs. LL+MM) | - | - | 1.16 (0.74-1.80) | 0.524 | 0.82 (0.43-1.55) | 0.530 |
| Recessive model  (MM vs. LL+LM) | - | - | 0.85 (0.50-1.47) | 0.570 | 2.78 (0.63-12.24) | 0.170 |
| Log-additive model | - | - | 1.16 (0.74-1.80) | 0.520 | 1.14 (0.68-1.91) | 0.620 |
| **Genotypes, genetic models *PON2* (S311C)** | | | | | | |
| SS | 48 (43.6%) | 85 (64.9%) | **Ref** | - | - | - |
| SC | 47 (42.7%) | 38 (29%) | 2.19 (1.26-3.82) | 0.006 | 2.52 (1.32-4.82) | 0.005 |
| CC | 15 (13.6%) | 8 (6.1%) | 3.32 (1.31-8.40) | 0.011 | 4.10 (1.46-11.55) | 0.008 |
| S allele frequency | 143 (65%) | 208 (79.4%) | **Ref** |  | - | - |
| C allele frequency | 77 (35%) | 54 (20.6%) | 2.70 (1.38-3.12) | 0.001 |  |  |
| Dominant model  (SS vs. SC+CC) | - | - | 2.39 (1.42-4.02) | 0.0009 | 2.79 (1.03-7.50) | 0.042 |
| Co-dominant model  (SC vs. SS+CC) | - | - | 1.83 (1.07-3.11) | 0.026 | 1.99 (1.08-3.68) | 0.028 |
| Recessive model  (CC vs. SS+SC) | - | - | 2.43 (0.99-5.96) | 0.047 | 2.78 (1.03-7.50) | 0.028 |
| Log-additive model | - | - | 1.96 (1.31-2.92) | 0.0007 | 2.19 (1.38-3.46) | 0.0006 |

SNPs: single nucleotide polymorphisms; OR= odds ratio; CI= confidence interval

^a^OR adjusted for age, BMI, WC, and hypertension

Bonferroni corrected p = 0.025 (p = 0.05 / number of SNPs)

**Supplementary Table 5. Distribution of clinical parameters in different genotypes of *PON1* (L55M) and *PON2* (S311C) in males**

|  | ***PON1* (L55M)** | | | | ***PON2* (S311C)** | | | |
| --- | --- | --- | --- | --- | --- | --- | --- | --- |
| **Variables** | **LL**  **(n=62)** | **LM**  **(n=34)** | **MM**  **(n=5)** | **p-value** | **SS**  **(n=38)** | **SC**  **(n=44)** | **CC**  **(n=19)** | **p-value** |
| **Age (years)** | 58.39±11.16 | 57.56±11.27 | 52±12.23 | 0.471 | 56.47±12.16 | 57.18±10.45 | 61.84±10.56 | 0.210 |
| **BMI (kg/m^2^)** | 26.33±4.17 | 27.13±4.24 | 24.14±2.28 | 0.285 | 27.25±4.29 | 25.91±3.55 | 26.31±4.79 | 0.341 |
| **WC (cm)** | 98.87±11.06 | 99.07±8.71 | 98.95±11.67 | 0.996 | 100.13±10.49 | 97.48±9.05 | 99.10±12.51 | 0.603 |
| **WHR** | 1.02±0.06 | 1.02±0.05 | 1.04±0.02 | 0.573 | 1.02±0.05 | 1.02±0.05 | 1.02±0.05 | 0.843 |
| **WHtR** | 0.58±0.06 | 0.59±0.05 | 0.59±0.06 | 0.847 | 0.59±0.06 | 0.58±0.05 | 0.58±0.07 | 0.445 |
| **SBP (mmHg)** | 132.48±18.41 | 135.75±17.97 | 141.87±22.29 | 0.444 | 133.02±18.01 | 135.15±21.10 | 133.50±12.52 | 0.866 |
| **DBP (mmHg)** | 84.78±10.67 | 83.12±9.74 | 89.99±14.09 | 0.374 | 83.92±9.91 | 86.12±11.49 | 81.79±9.22 | 0.302 |
| **PP** | 47.71±10.61 | 52.64±13.14 | 51.87±8.72 | 0.122 | 49.10±12.75 | 49.04±12.12 | 51.72±7.38 | 0.672 |
| **TC (mg/dL)** | 292.24±35.02 | 301.51±51.62 | 285.52±22.85 | 0.498 | 302.29±49.44 | 287.97±30.43 | 296.83±42.64 | 0.283 |
| **TG (mg/dL)** | 222.84±67.19 | 217.41±54.67 | 279.34±76.94 | 0.131 | 232.28±65.40 | 212.68±57.59 | 232.63±76.08 | 0.315 |
| **HDL-C (mg/dL)** | 24.03±4.71 | 23.43±5.12 | 24.69±5.55 | 0.786 | 22.75±4.74 | 24.24±4.66 | 25.19±5.29 | 0.156 |
| **LDL-C (mg/dL)** | 222.55±38.36 | 230.36±41.71 | 204.96±32.98 | 0.347 | 231.32±47.32 | 219.43±31.28 | 221.55±38.62 | 0.376 |
| **VLDL-C (mg/dL)** | 45.82±16.94 | 47.72±22.62 | 55.87±15.39 | 0.504 | 48.22±17.82 | 44.29±17.59 | 50.61±23.72 | 0.421 |

BMI: body mass index; WC: waist circumference; WHR: waist-to-hip ratio; WHtR: waist-to-height ratio; SBP: systolic blood pressure; DBP: diastolic blood pressure; PP: pulse pressure; TC: total cholesterol; TG: triglycerides; HDL-C: high-density lipoprotein-cholesterol; LDL-C: low-density lipoprotein cholesterol; VLDL-C: very-low-density lipoprotein cholesterol; Bonferroni corrected p=0.003

**Supplementary Table 6. Distribution of clinical parameters in different genotypes of *PON1* (L55M) and *PON2* (S311C) in females**

|  | ***PON1* (L55M)** | | | | ***PON2* (S311C)** | | | |
| --- | --- | --- | --- | --- | --- | --- | --- | --- |
| **Variables** | **LL**  **(n=69)** | **LM**  **(n=34)** | **MM**  **(n=7)** | **p-value** | **SS**  **(n=48)** | **SC**  **(n=47)** | **CC**  **(n=15)** | **p-value** |
| **Age (years)** | 56.35±11.28 | 59.21±11.31 | 66.57±11.43 | 0.057 | 56.67±10.12 | 59.28±13.26 | 57.40±9.70 | 0.538 |
| **BMI (kg/m^2^)** | 26.66±5.18 | 27.92±6.34 | 29.37±5.95 | 0.328 | 26.52±6.32 | 27.78±5.21 | 27.69±4.39 | 0.520 |
| **WC (cm)** | 98.55±10.27 | 102.21±12.26 | 105.26±11.48 | 0.130 | 98.99±12.16 | 101.77±10.27 | 98.47±9.94 | 0.395 |
| **WHR** | 1.00±0.06 | 1.02±0.06 | 0.99±0.05 | 0.114 | 1.02±0.06 | 1.01±0.06 | 0.99±0.06 | 0.543 |
| **WHtR** | 0.62±0.06 | 0.64±0.09 | 0.67±0.08 | 0.137 | 0.63±0.08 | 0.64±0.07 | 0.62±0.06 | 0.576 |
| **SBP (mmHg)** | 134.01±18.84 | 133.65±15.63 | 126.14±15.75 | 0.535 | 134.87±15.33 | 132.48±20.34 | 131.51±16.49 | 0.733 |
| **DBP (mmHg)** | 85.49±12.11 | 86.51±9.07 | 83.81±8.34 | 0.815 | 85.62±10.33 | 85.35±10.63 | 86.98±14.44 | 0.886 |
| **PP** | 48.51±12.22 | 47.14±10.26 | 42.33±10.12 | 0.383 | 49.26±9.78 | 47.11±13.1 | 44.53±11.09 | 0.348 |
| **TC (mg/dL)** | 280.76±28.92 | 279.11±25.95 | 301.95±30.80 | 0.141 | 281.83±30.16 | 281.00±28.42 | 282.68±23.94 | 0.978 |
| **TG (mg/dL)** | 246.92±83.98 | 217.03±47.34 | 213.79±40.54 | 0.108 | 243.15±71.44 | 231.77±80.63 | 223.22±55.19 | 0.592 |
| **HDL-C (mg/dL)** | 25.52±5.54 | 24.96±5.99 | 27.34±3.39 | 0.591 | 26.13±4.98 | 24.59±6.08 | 26.35±5.63 | 0.369 |
| **LDL-C (mg/dL)** | 205.28±35.22 | 210.74±31.58 | 231.86±30.51 | 0.134 | 206.34±34.98 | 210.05±35.23 | 211.69±29.85 | 0.815 |
| **VLDL-C (mg/dL)** | 49.96±17.95 | 43.41±9.47 | 42.76±8.12 | 0.093 | 49.46±16.22 | 46.36±16.13 | 44.64±11.04 | 0.471 |

BMI: body mass index; WC: waist circumference; WHR: waist-to-hip ratio; WHtR: waist-to-height ratio; SBP: systolic blood pressure; DBP: diastolic blood pressure; PP: pulse pressure; TC: total cholesterol; TG: triglycerides; HDL-C: high-density lipoprotein-cholesterol; LDL-C: low-density lipoprotein cholesterol; VLDL-C: very-low-density lipoprotein cholesterol; Bonferroni corrected p=0.003

**Supplementary Table 7. Haplotype analysis of *PON1* (L55M) and *PON2* (S311C) polymorphisms in the male and female study participants**

| **Males** | | | | | | |
| --- | --- | --- | --- | --- | --- | --- |
|  | ***PON1*** | ***PON2*** | **OR (95%CI)** | **p-value** | **^a^OR (95%CI)** | **p-value** |
| 1 | L | S | 1.00 | -- | 1.00 | -- |
| 2 | L | C | 2.52 (1.54-4.13) | 0.0003 | 2.21 (1.29-3.80) | 0.004 |
| 3 | M | S | 1.61 (0.89-2.90) | 0.120 | 1.33 (0.69-2.53) | 0.390 |
| 4 | M | C | 0.97 (0.33-2.86) | 0.950 | 1.26 (0.43-3.73) | 0.680 |
| Global Haplotype association p-value=0.001 | | | | | | |
| **Females** | | | | | | |
|  | ***PON1*** | ***PON2*** | **OR (95%CI)** | **p-value** | **^b^OR (95%CI)** | **p-value** |
| 1 | L | S | 1.00 | -- | 1.00 | -- |
| 2 | L | C | 2.22 (1.34-3.67) | 0.002 | 2.41 (1.36-4.27) | 0.003 |
| 3 | M | S | 1.39 (0.77-2.49) | 0.280 | 1.32 (0.66-2.64) | 0.430 |
| 4 | M | C | 1.75 (0.67-4.56) | 0.250 | 2.09 (0.77-5.69) | 0.150 |
| Global Haplotype association p-value=0.006 | | | | | | |

OR: odds ratio, CI: confidence interval

^a^OR adjusted for age, BMI, WC, and hypertension and alcohol consumption

^b^OR adjusted for age, BMI, WC, and hypertension

Bonferroni corrected p = 0.0125 (p-value = 0.05/number of haplotypes)
